# Single nephron glomerular filtration rate measured by linescan multiphoton microscopy compared to conventional micropuncture

**DOI:** 10.1007/s00424-022-02686-8

**Published:** 2022-04-09

**Authors:** Vincenzo Costanzo, Luciano D’Apolito, Donato Sardella, Anna Iervolino, Gaetano La Manna, Giovambattista Capasso, Sebastian Frische, Francesco Trepiccione

**Affiliations:** 1 Department of Experimental, Diagnostic and Specialty Medicine, University of Bologna, Bologna, Italy; 2 Biogem, Institute of Molecular Biology and Genetics, Ariano Irpino, Italy; 3 Department of Medical Translational Sciences, University of Campania “Luigi Vanvitelli”, Via Pansini n5, 80131 Naples, Italy; 4 Department of Biomedicine, Aarhus University, Aarhus, Denmark

**Keywords:** Micropuncture, Single nephron glomerular filtration rate, Linescan, Multiphoton microscopy, Kidney physiology, Ischemia/reperfusion

## Abstract

Renal micropuncture, which requires the direct access to the renal tubules, has for long time been the technique of choice to measure the single nephron glomerular filtration rate (SNGFR) in animal models. This approach is challenging by virtue of complex animal preparation and numerous technically difficult steps. The introduction of intravital multiphoton microscopy (MPM) offers another approach to the measure of the SNGFR by mean of the high laser-tissue penetration and the optical sectioning capacity. Previous MPM studies measuring SNGFR *in vivo* relied on fast full-frame acquisition during the filtration process obtainable with high performance resonant scanners. In this study, we describe an innovative linescan–based MPM method. The new method can discriminate SNGFR variations both in conditions of low and high glomerular filtration, and shows results comparable to conventional micropuncture both for rats and mice. Moreover, this novel approach has improved spatial and time resolution and is faster than previous methods, thus enabling the investigation of SNGFR from more tubules and improving options for data-analysis.

**Supplementary Information:**

The online version contains supplementary material available at 10.1007/s00424-022-02686-8.

## Introduction

The single nephron glomerular filtration rate (SNGFR) refers to the rate of ultrafiltrate production measured at a level of a single glomerulus, showing a precise measure of the glomerular dynamics [36].

SNGFR changes are associated to specific body conditions, including extracellular fluid volume expansion [3] and dietary proteins intake [20], and to pathological states, like obesity [7] and/or diabetes mellitus [33].

For a long time, micropuncture approaches have been used to calculate SNGFR in animal models. According to the standard formula, SNGFR assessment requires the evaluation of inulin in the blood and in a fluid sample in ratio with the tubular fluid flow rate [18]. Micropuncture has been the technique of choice to investigate SNGFR, despite the need for a very complex animal preparation and a sophisticated equipment to carry out the experiments. Additionally, micropuncture is limited to conventional microscopy, which allows to image only a few microns under the renal capsule providing limited access to deeper renal structures [21].

The advent of multiphoton microscopy (MPM) facilitated *in vivo* imaging of physiological processes compared to other imaging techniques due to improved laser penetration and less light scattering. Unlike the conventional one photon fluorescence, MPM uses a long-wavelength laser in the range of near infrared (700–1000 nm) and 2 photons arriving simultaneously on the sample. The resulting lower light dispersion and low energy of the photons lead to deeper tissue imaging and less phototoxicity. Since the excitation energy tends to drastically decrease by going far from the focal plane, photobleaching and out of plane signal are much reduced [37].

Because of deeper penetration in renal parenchyma, MPM allowed the visualization of structures like the juxta-glomerulus apparatus and the investigation of physiological processes, including the tubule-glomerular feedback (TGF) and renin release [8, 25] as well as the proximal tubule endocytosis and the vascular permeability [30].

Additionally, MPM enables the detection of the autofluorescence naturally emitted by the kidney, hence allowing to recognize the renal structures without external labeling. In fact, nicotinamide adenine dinucleotide (NADH) fluorescence naturally exhibited in its reduced state by mitochondria and lysosomes allows to easily recognize the proximal tubules where these organelles are abundant, while other segments of the nephron such as distal tubules and collecting ducts appear as less evident structures. Superficial glomeruli lack any fluorescence and appear as large dark empty spaces close to proximal tubules [30].

The renal autofluorescence is also helpful to investigate the mitochondrial function and metabolic state of the cells, as previously shown [12].

The main advantage of MPM is the possibility to label many cellular structures by using concurrently up to 4–5 different fluorescent markers, allowing multiple comparisons of labeled probes and simultaneous analysis of different parameters [30].

An interesting application of MPM was developed by Kang et al. [15] for *in vivo* SNGFR measurements. The authors used resonant scanners to acquire a full-frame time-series of the field of view at high temporal resolution (20 Hz). This allowed the necessary temporal and spatial resolution to record the entire glomerular filtration process of a fluorescent dye, from the vascular perfusion of the glomerular capillaries to the diffusion in the Bowman space and tubular lumen.

However, in order to achieve SNGFR measurement in the absence of high-performance resonant scanners, we develop here a new MPM imaging approach based on linescan acquisition, whereby high-performance galvanometers direct the laser beam along a predefined freehand drawn line at very high temporal resolution (> 400 Hz).

## Methods

### Multiphoton microscopy

MPM was performed using an upright Ultima Investigator 2-photon microscope (Bruker, MS, USA) equipped with a water-immersion 20 × objective XLUMPlanFL20XW NA 1.0, (Olympus, Japan) and supplemented by a converter arm (Inverterscope, LSM TECH, USA) to allow inverted imaging. The Ti–Sapphire laser (Mai Tai® DeepSee™, Spectra-Physics, USA) was tuned for an excitation wavelength of 800 nm. For the detection of FITC (green channel), emitted light between 500 and 550 nm was recorded using a Hamamatsu model H10770PB-40 GaAsP-detector. For the detection of TRITC (red channel) and autofluorescence (blue channel) light between 570 and 620 and between 435 and 485 nm, respectively, were recorded using a Hamamatsu model R3896 multi-alkali detectors. The images were collected at 512 × 512 resolution, and the pixel dwell time was 2.8 µs. The microscope was controlled by Prairie View software.

### Animal experiment

Female (170–220 g) and male (260–295 g) Munich-Wistar Frömter (MWF) rats, and male (20–30 g) C57BL/6 mice were used in this study. After anesthesia with ip injection of thiobutabarbital for rats (Inactin, 120-mg/kg body weight) and tiletamine hydrochloride and zolazepam hydrochloride (Zoletil, 40-mg/kg body weight) and xylazine hydrochloride (Rompun, 4-mg/kg body weight) for mice, the trachea was cannulated with a polyethylene catheter (PE210 for rats, PE90 for mice, 2Biological Instruments) to assist breathing, and the right jugular vein and the left carotid artery were cannulated with a polyethylene catheter (PE50 for rats, PE10 for mice, 2Biological Instruments).

The catheter in the jugular vein of rats was Y shaped in order to ensure a simultaneous injection of drugs and the fluorescent markers. Finally, the left kidney was exteriorized through a 10–15-mm flank incision. The animals were placed on the stage of an inverted microscope with the exteriorized kidney placed in a coverslip-bottomed cell culture dish rinsed in warm 0.9% saline solution (Fig S1).

All the procedures were performed on a thermic pad (Kent Scientific) in order to keep the body temperature constant at 37 °C.

During the *in vivo* imaging, arterial blood pressure and heart rate were continuously monitored by mean of a pressure transducer (BP-1, 2Biological Instruments), connected to a power lab system (ADinstruments, 2Biological Instruments), as shown previously [14]. Blood gas analysis was evaluated by Epoc blood system analysis (Siemens) during the experiment, as previously done [34].

### Ischemia-reperfusion injury

For the induction of ischemia–reperfusion injury (IRI), after kidney externalization, the renal pedicle was clamped for 30 min, then the clamp was released to allow blood to reperfuse the kidney. SNGFR was measured during the 120-min reperfusion period. For IRI experiments, 3 female MWF rats (185–205 g) were used.

### Dopamine infusion

Dopamine hydrochloride (S.A.L.F. Spa) was diluted in normal 0.9% saline and given IV at low dosage of 3 μg/kg/min in continuous infusion (20 μl/min). The infusion of dopamine started 2 min after the beginning of the imaging and lasted until the end of the experiment. For control animals, a continuous IV infusion (20 μl/min) of normal 0.9% saline was administered during the experiments. The injection of fluorescent probes, and drug was carried out using an automatic infusion pump (KD Scientific, 2Biological Instruments). For dopamine infusion experiments, 3 female MWF rats (176–197 g) were used.

### Fluorescent probes and drugs

The 500-kDa tetramethylrhodamine isothiocyanate–dextran (TRITC, 52194-1G, sigma) was used to label peritubular and glomerular capillaries (150 μl for rats, 50 μl for mice of a 10-mg/ml stock IV bolus). The freely filtered 3–5-kDa fluorescein isothiocyanate–dextran (FITC, FD4-1G, sigma) was used as a tracer molecule to measure the filtration rate (30 μl for rats, 10 μl for mice of a 10-mg/ml stock IV bolus).

### Statistical analysis

Figures and data analysis were performed using Fiji and Graph Pad Prism 7 software. The values are expressed as mean ± SEM. Statistical analysis was performed by one-way ANOVA followed by the Tukey’s multiple comparison test or by *t*-test for SNGFR measurements considering single values of SNGFR for each group. *P* values < 0.05 were considered statistically significant.

## Results

### Linescan-used method allows SNGFR evaluation at low full-frame acquisition

**Fig. 1:**
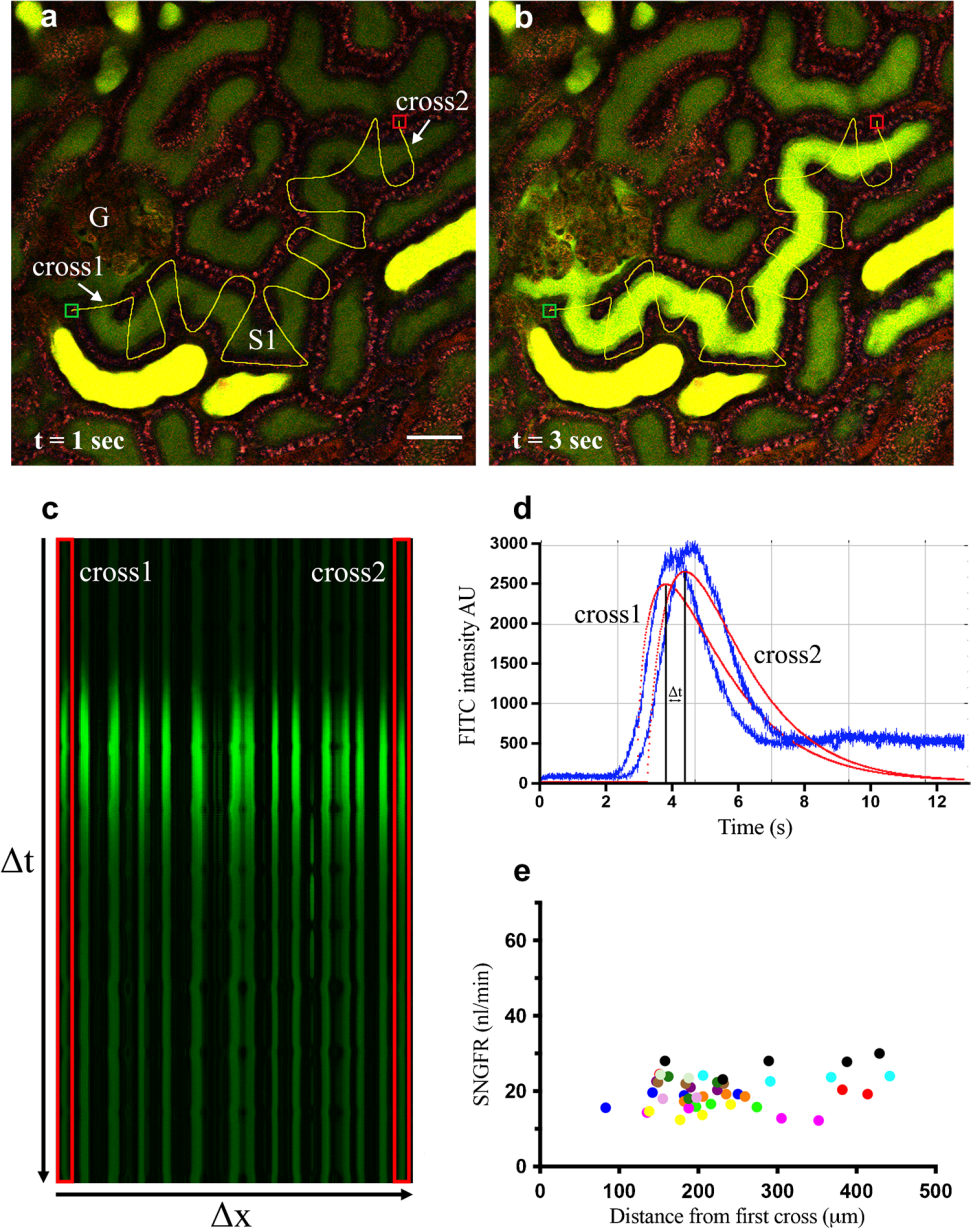
Linescan method for *in vivo* SNGFR measurements. Panels **a** and **b** show the glomerular filtration of low-molecular weight FITC-dextran (3–5 kDa) during the linescan acquisition. Multiple crossings are hand-drawn perpendicularly to the tubular lumen and acquired while the fluorescent dextran is injected. FITC 3–5 kDa (green) is freely filtered through the glomerulus (G) and streams along the tubular lumen of early proximal tubule (S1). *t* represents the time in seconds after bolus injection. Scale bar is 50 μm. In panel **c**, each fluorescent line corresponds to a tubular crossing. Red selected areas indicate the two tubular crosses used for the analysis. In panel **d**, FITC 3–5-kDa fluorescence intensity as arbitrary unit (AU) acquired at red selected areas is plotted over time. The blue curves represent the original intensity plots, while the fitted curves are shown in red. Panel **e** shows SNGFR as calculated in the same tubules (individual colors) at different distances between the two crosses (45 measurements from 12 different tubules)

For measurements of SNGFR, glomeruli connected to S1 proximal tubules extending for at least 100 μm from the exit of the Bowman’s space were identified. A linescan path starting from the urinary pole and crossing several times the tubular lumen in an orthogonal manner was hand drawn (Fig. 1a, b). In the setting of the linescan-acquisition parameters, an average of 7000 scan per line were usually set up with an average linescan period of 2.23 ms (448 Hz) resulting in a total scan time of about 15 s. These parameters are usually enough to efficiently trace the filtration of 30-μl IV bolus of FITC-dextran 3–5 kDa through the glomerulus. The linescan was acquired soon after the IV bolus of FITC 3–5-kDa conjugated marker was injected.

After the acquisition, the single pictures of each linescans were combined together in a single *x*–*t* (space vs time) image from Prairie View software. The x axis of this path corresponds to the length of the drawn line (Δ*x*), while the y axis to the scan time (Δ*t*) (Fig. 1c). In the merged picture, the crosses of the fluorescent dye with the linescan path are represented by fluorescent vertical lines with a segment of higher intensity that is progressively shifting down along the y axis. On the other hand, the linescan path between tubular lumen crossing is not showing any fluorescence (black color) (Fig. 1c).

In order to evaluate the speed by which the fluorescent signal moves, two lines of interest (called cross1 and cross2 in Fig. 1a), were chosen among the several crossing of the linescan. One was always selected as the closest to the urinary pole, while the other one was generally chosen at the end of the linescan path (at least 100 μm downstream along the S1 segment of the proximal tubule).

The fluorescence analysis at the two crosses of interest was then derived by selecting the area containing the two lines (red rectangles) and plotting the fluorescence intensity profile over time in Fiji [31], as showed in Fig. 1d. The resulting curves were fitted by using the gamma variate function in Fiji, in order to ensure a clearer fluorescent intensity signal. The difference in the time required by the same dye to reach the two selected crosses of interest (Δ*T*) was calculated as the time difference between the two peaks of fluorescence intensity (Fig. 1d). Finally, SNGFR was calculated from the data measured as the fluid volume that moves between the two crosses in the time frame Δ*T* (tubular length × (diameter/2)^2^ × π × Δ*T*). The tubular length within the crosses of interest and the average tubular diameter were properly measured in Fiji (Fig.  S2).

Due to the multiple tubular crossing drawn with linescan tool, this method allows to obtain several SNGFR measurements of the same tubule and to test the consistency of the analysis by investigating changes in flow rate along the tubule due to fluid reabsorption. This is accomplished by changing the cross2 every time the analysis is performed. As showed in Fig. 1e, the SNGFR values remained consistent in respect to the distance between the 2 crosses, suggesting that tubular reabsorption of the fluorescent dextran or water along the proximal tubule is negligible in this setting. In order to exclude a possible tubular reabsorption, we measured the fluorescence intensity over time in 7 regions of interest (ROIs) of the cellular compartment of S1 proximal tubule during the bolus injection of FITC 3–5 kDa (Fig. S3a). As shown in Fig. S3b, the curves remain stable over time during the FITC 3–5-kDa bolus. This indicates that no acquisition of the intracellular fluorescent signal over the time.

Furthermore, this experimental approach allows to inject a second bolus of the fluorescent dye in the same tubule after wash-out of the first FITC 3–5-kDa bolus. In this way, intra-nephron variability over time can be evaluated. As showed in Fig. S4, SNGFR values of the same tubule obtained after two consecutive bolus injections are consistent, showing no significant difference between the two measurements.

### SNGFR evaluation in context of low and high glomerular filtration

**Fig. 2:**
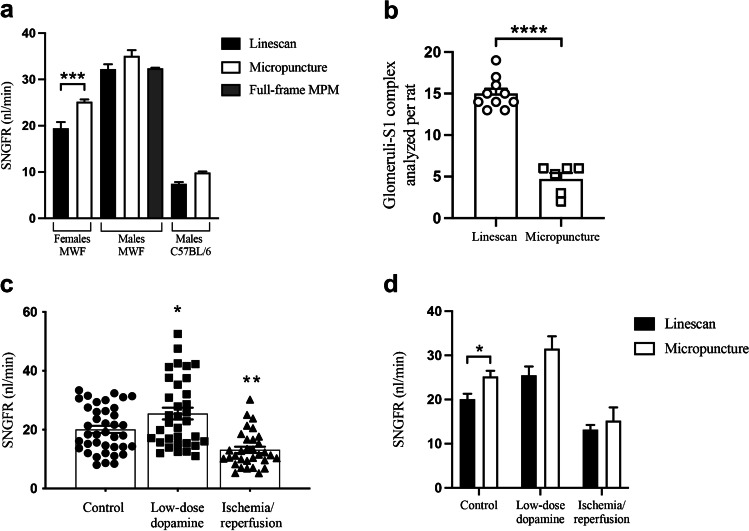
SNGFR measurement with linescan method. In panel **a**, SNGFR values evaluated by linescan method (black square) were compared with data obtained by full-frame MPM (gray square) [15] and micropuncture studies [23, 32] (white square). SNGFR measurements in mice were compared with micropuncture data [19]. *** is for *p*-value < 0.001 (unpaired *t*-test). For male rats one-way ANOVA followed by Tukey’s multiple comparison test was used. In panel **b**, the average number of glomeruli-S1 complex per rat acquired with linescan or micropuncture ([4, 11, 24, 27, 28, 35]) is reported (15 ± 0.6 from 10 rats versus 4.7 ± 0.72 from 6 different experimental studies). **** is for *p*-value < 0.0001 (unpaired *t* test). In panel **c**, the distribution of the SNGFR values per single glomerulus evaluated by linescan tool is measured at control, low dose dopamine infusion and after IRI. * is for *p*-value < 0.05 and ** is for *p*-value < 0.01 versus the control group (one-way ANOVA, followed by Tukey’s multiple comparison test). In panel **d**, the mean SNGFR values evaluated at control, low-dose dopamine infusion and IRI with linescan method or micropuncture were compared. * is for *p*-value < 0.05 (unpaired *t* test). All the data are expressed as mean ± standard error

We first aimed to assess the SNGFR in control MWF rats to test the feasibility of the linescan approach. SNGFR averaged 19.43 ± 2.36 and 32.21 ± 2.38 nl/min in female and male rats, respectively (Fig. 2a). We used younger females compared with the ones reported in [23] (Tab-S1), this likely accounts for the significant lower SNGFR detected by the linescan method. Data from male rats were more consistent with data from the literature evaluated both with micropuncture [32] and MPM [15]. This method can be applied also to proximal tubules from mice with values similar to micropuncture [19] (7.45 ± 0.65 vs 9.9 ± 0.6 nl/min) (Fig. 2a).

Compared to micropuncture the linescan approach allows the measurement of a larger number of glomeruli per rat, as indicated in Fig. 2b.

**Fig. 3:**
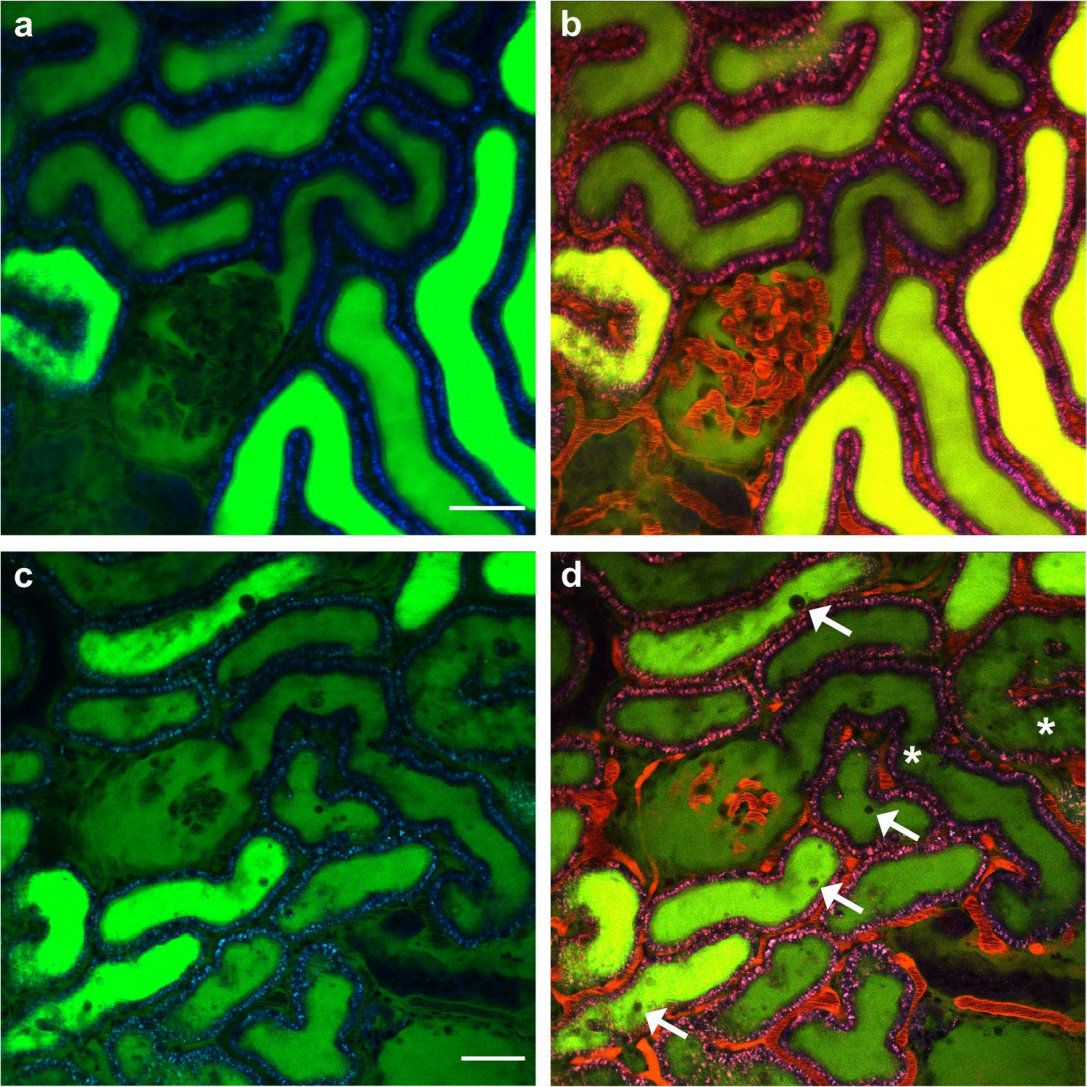
Visualization of healthy and ischemic rat kidney. Representative images from control MWF rats (panels **a** and **b**) and IRI-treated rats (panels **c** and **d**). Renal vasculature is labeled with TRITC-dextran 500 kDa (red), while kidney autofluorescence appears in blue. Tubular damage occurs after 30 min from IRI. Altered tubular morphology (asterisks) and intraluminal debris (arrows) appear during the reperfusion phase. Panels b and d include the vasculature. Scale bar is 50 μm

In order to investigate whether the linescan method is reliable to detect variation of SNGFR from normal values, we studied the model of IRI to recapitulate AKI [2] and low-dose dopamine to mimic known condition of hyperfiltration (increased SNGFR) [22]. In female rats with IRI, the SNGFR was significantly reduced compared to the controls (13.17 ± 1.06 vs 19.43 ± 2.36 nl/min. (Fig. 2c, d). On the same line, we were able to distinguish a significant increase in SNGFR after injection of low-dose dopamine (25.48 ± 2 nl/min vs 19.43 ± 2.36 nl/min. (Fig. 2c, d). The variability of the SNGFR within the single animal is reported in Fig. 5S. Lower SNGFR after IRI was associated with severe morphological alteration as tubular necrosis, intraluminal cast and debris formation, and vascular congestion (Fig. 3) as consistent with previous data [1].

## Discussion

Micropuncture has been the only method to evaluate SNGFR for decades. This approach provided reliable and accurate measurements of SNGFR, although it is a very laborious technique [21]. The advances of *in vivo* microscopy provide real-time high-resolution images of deep kidney structures and 3D movies of complex renal processes in health and disease [8, 30] as well as morphological label-free evaluations of kidney sections [26]. Among the several applications, the assessment of SNGFR by MPM was established by Kang et al. [15], then recently expanded by Kessel and collaborators [16], who obtained reliable values by exploiting the multiphoton full-frame acquisition during the filtration process. This fast imaging modality relied on the usage of resonant scanners, which represents an optional equipment able to reach very high acquisition speed in comparison to usual MPM. Indeed, the dynamic cellular processes, especially when investigated in living tissues, require high performance in terms of acquisition speed to avoid loss of information. The resonant scanners exploit a single-turn coil in the scanning system that moves allowing it to vibrate at very high frequency [5]. The resulting acquisition speed is increased in comparison to conventional MPM, and it allows to study very dynamic events such as metastatic cell movement within brain vessels and cell flow in lymphatic circulation [17]. Nevertheless, as Dunn and colleagues stated, the advantage shown by resonant scanners not necessarily is helpful to study *in vivo* kidney function. In fact, traditional galvanometric scanners are usually fast enough to image most renal processes [9].

However, not all the microscopes are equipped with resonance scanners, and their implementation would lead to an additional cost. Furthermore, the achievement of such a speed may considerably reduce the signal to background ratio, making the imaging noisier, especially when single cells are tracked. This, in turn, would force investigators to use filters and imaging software to improve the image quality [17].

In order to overcome this limitation, to measure SNGFR, we set up a method based on linescan imaging acquisition, which allows to acquire repetitive scans with very high temporal resolution within ROI. The linescan approach has been previously used on kidneys to evaluate *in vivo* red blood cell velocity in capillaries [10, 15, 30].

Linescan tool does not require additional microscope equipment, and it permits to considerably increase the frame rate compared to resonant scanners. In fact, the acquisition by laser beam is only focused along the traced lines instead of the entire field of view. The fast acquisition offered by linescan method allows to measure in the same animal more tubules over time compared to full-frame technique. Additionally, with linescan method more data points along each tubule can be obtained. This gives better signal/noise ratio for changes in fluorescence signal along the tubule, which in turn provides a better possibility to evaluate water or dextran tubular reabsorption more precisely. Moreover, the output of the linescan is an image rather than a movie, and this reduces the size of data storage compared to full-frame acquisition, since the structures not included in the drawn lines are not acquired.

The linescan tool used for SNGFR evaluation ensures the precision of the measurement at different distances from the glomerulus at the same extent as shown by Kang et al. [15], and it provides comparable values of SNGFR as obtained by previous micropuncture experiments in gender/aged-matched rats [23, 32] and in mice [19]. The small variance found in SNGFR value between the two approaches could be due to the slight difference in rats’ age [13]. The results we obtained by linescan were also very similar to previously reported data from MPM [15] (Fig. 2a).

We validated this method also in the experimental setting of hyperfiltration as by low-dose dopamine infusion. Dopamine at lower dosage (< 5 μg/kg/min) raises SNGFR by specifically vasodilating the renal artery as described before [6, 32]. In fact, the SNGFR measurements we achieved with the linescan method in dopamine-treated rats were significantly increased compared to controls, and these values were comparable to those measured by micropuncture [29] (Fig. 2d).

On the same line, we tested the linescan method after IRI, in order to detect a reduction in SNGFR. The approach we used to induce ischemic condition provides a good option to mimic the human acute renal injury in rodents [12]. In agreement with previous studies [1], we observed during the reperfusion phase variable tubular necrosis characterized by loss of the proximal tubule morphology, and intraluminal cast and cellular debris formation due to shedding of the cellular components in the tubular lumen. In addition, the renal blood vessels appeared variably constricted and the resulting renal blood flow was reduced as consequences of vascular endothelium damages. After 30 min of ischemia, the rats showed a significant reduction of SNGFR, consistently with previous micropuncture experiments [1] (Fig. 2d).

Therefore, the imaging method we developed could be used as fast screening tool to evaluate the effects of single molecules and specific physiological conditions (including acid–base alteration and electrolyte imbalance) on the SNGFR.

The comparison of the experimental setting used in our linescan tool and in previous micropuncture experiments is listed in Table S1.

MPM did not differ from a micropuncture-based approach for the type of glomeruli to be accessible. Indeed, both techniques can access only superficial glomeruli. For this reason, for the experiments of dopamine infusion and IRI, we used female MWF rats that present with a large number of superficial glomeruli compared to males [28]. In addition, male MWF rats develop massive and progressive proteinuria already at 7 weeks of age [28]. However, previous studies using micropuncture have analyzed on average not more than 5 or 6 glomeruli per animal when SNGFR is investigated, due to technical issues [4, 11, 24, 27, 28, 35]. In contrast, MPM approach allows the evaluation of three times more glomeruli, thus increasing the reliability of the analysis.

Taken together, this study shows that the linescan-based MPM method is reliable and easy to apply to measure SNGFR in health and disease in rats and mice, representing a promising alternative to the more challenging micropuncture. Moreover, compared to previous MPM studies that assess SNGFR using full-frame acquisition, the linescan-based method requires a cheaper microscopy set-up.

## Supplementary Information

Fig S1 Experimental setup for *in vivo* MPM imaging of the kidney. Anesthetized animals were surgically prepared for intravital imaging as described in methods. An inverted arm was used to acquire the images in order to minimize the movements due to breathing. A water-immersion 20X NA 1.0 objective was used to image the renal structures. The animal was covered by a warm pad for the entire experimental procedure. (PNG 801 kb)

High Resolution Image (TIF 2596 kb)

Fig S2 Measurement of tubular length and diameter of S1 proximal tubule. Representative image of the S1 proximal tubule after the linescan acquisition. The tubular length was measured within the two crosses of interest by following the central axis of the lumen. The tubular diameter was measured in several points along the tubule, then all measurements were averaged to measure the SNGFR. Fiji software was used to analyze the images. Scale bar is 50 μm (PNG 991 kb)

High Resolution Image (TIF 3970 kb)

Fig S3 Analysis of intracellular fluorescence during dextran bolus administration. Seven ROIs were selected in the intracellular compartment of S1 proximal tubule (panel a) and the variation of fluorescence intensity expressed as arbitrary unit intensity (AU) was recorded over the time the bolus injection of FITC-dextran 3-5 kDa streamed along the tubule (panel b). Each ROI is represented by a different color. Scale bar is 50 μm. (PNG 1420 kb)

High Resolution Image (TIF 6327 kb)

Fig S4 Consistency of the SNGFR over time along the same S1 proximal tubule. SNGFR was measured two consecutive times in nine S1 proximal tubules from different animals. In particular, a second bolus of the fluorescent dye (bolus 2) was performed in the same tubule within 5-10 minutes after the first one was injected (bolus 1). No difference of SNGFR values after bolus 1 and bolus 2 was detected (Paired t test). (PNG 33 kb)

High Resolution Image (TIF 118 kb)

Fig S5 SNGFR per single S1 segment at control, low dose dopamine and IRI. Single data from each S1 segment are plotted. Each bar represents measurements from one experimental rat. Mean values ± standard error are represented. (PNG 53 kb)

High Resolution Image (TIF 215 kb)

Table S1 Comparison of the experimental setting used in MPM linescan tool and previous micropuncture studies. The table compares the physiological parameters of rats used for experiments for linescan method and previous micropuncture. In particular, the age (days) and the body weight (g) of the animals and the references are reported. (DOCX 15 kb)

## References

[1] Bird JE, Milhoan K, Wilson CB, Young SG, Mundy CA, Parthasarathy S, Blantz RC (1988) Ischemic acute renal failure and antioxidant therapy in the rat. The relation between glomerular and tubular dysfunction. J Clin Invest 81:1630–1638. http://www.jci.org/articles/view/113498 10.1172/JCI113498 PMC442599 2835399

[2] Bonventre J V., Yang L (2011) Cellular pathophysiology of ischemic acute kidney injury. J Clin Invest 121:4210–4221. http://www.jci.org/articles/view/45161 10.1172/JCI45161 PMC3204829 22045571

[3] Brenner B, Daugharty T, Ueki I, Troy J (1971) Quantitative assessment of proximal tubule function in single nephrons of the rat kidney. Am J Physiol Content 220:2058–2067. https://www.physiology.org/doi/10.1152/ajplegacy.1971.220.6.2058 10.1152/ajplegacy.1971.220.6.2058 5087858

[4] Brenner BM, Troy JL, Daugharty TM (1971) The dynamics of glomerular ultrafiltration in the rat. J Clin Invest 50:1776–80. http://www.ncbi.nlm.nih.gov/pubmed/5097578 10.1172/JCI106667 PMC442078 5097578

[5] Carriles R, Schafer DN, Sheetz KE, Field JJ, Cisek R, Barzda V, Sylvester AW, Squier JA (2009) Invited review article: imaging techniques for harmonic and multiphoton absorption fluorescence microscopy. Rev Sci Instrum 80:081101. http://www.ncbi.nlm.nih.gov/pubmed/19725639 10.1063/1.3184828 PMC2736611 19725639

[6] Choi MR (2015) Renal dopaminergic system: pathophysiological implications and clinical perspectives. World J Nephrol 4:196. http://www.wjgnet.com/2220-6124/full/v4/i2/196.htm 10.5527/wjn.v4.i2.196 PMC4419129 25949933

[7] Denic A, Mathew J, Lerman LO, Lieske JC, Larson JJ, Alexander MP, Poggio E, Glassock RJ, Rule AD (2017) Single-nephron glomerular filtration rate in healthy adults. N Engl J Med 376:2349–2357. http://www.nejm.org/doi/10.1056/NEJMoa1614329 10.1056/NEJMoa1614329 PMC5664219 28614683

[8] Dunn KW, Sandoval RM, Kelly KJ, Dagher PC, Tanner GA, Atkinson SJ, Bacallao RL, Molitoris BA (2002) Functional studies of the kidney of living animals using multicolor two-photon microscopy. Am J Physiol Physiol 283:C905–C916. https://www.physiology.org/doi/10.1152/ajpcell.00159.2002 10.1152/ajpcell.00159.2002 12176747

[9] Dunn KW, Sutton TA, Sandoval RM (2018) Live-animal imaging of renal function by multiphoton microscopy. Curr Protoc Cytom 83:12.9.1–12.9.25. http://www.ncbi.nlm.nih.gov/pubmed/29345326 10.1002/cpcy.32 PMC5774340 29345326

[10] Ferrell N, Sandoval RM, Bian A, Campos-Bilderback SB, Molitoris BA, Fissell WH (2015) Shear stress is normalized in glomerular capillaries following 5/6 nephrectomy. Am J Physiol Physiol 308:F588–F593. https://www.physiology.org/doi/10.1152/ajprenal.00290.2014 10.1152/ajprenal.00290.2014 PMC4360039 25587117

[11] Fogo A, Yoshida Y, Glick AD, Homma T, Ichikawa I (1988) Serial micropuncture analysis of glomerular function in two rat models of glomerular sclerosis. J Clin Invest 82:322–30. http://www.ncbi.nlm.nih.gov/pubmed/3392211 10.1172/JCI113590 PMC303512 3392211

[12] Hall AM, Molitoris BA (2014) Dynamic multiphoton microscopy: focusing light on acute kidney injury. Physiology 29:334–342. https://www.physiology.org/doi/10.1152/physiol.00010.2014 10.1152/physiol.00010.2014 PMC4214830 25180263

[13] Horster M, Valtin H (1971) Postnatal development of renal function: micropuncture and clearance studies in the dog. J Clin Invest 50:779–795. http://www.jci.org/articles/view/106549 10.1172/JCI106549 PMC291992 5547275

[14] Iervolino A, Trepiccione F, Petrillo F, Spagnuolo M, Scarfò M, Frezzetti D, De Vita G, De Felice M, Capasso G (2015) Selective dicer suppression in the kidney alters GSK3β/β-catenin pathways promoting a glomerulocystic disease. PLoS One 10:e0119142. http://www.ncbi.nlm.nih.gov/pubmed/25799508 10.1371/journal.pone.0119142 PMC4370407 25799508

[15] Kang JJ, Toma I, Sipos A, McCulloch F, Peti-Peterdi J (2006) Quantitative imaging of basic functions in renal (patho)physiology. Am J Physiol Physiol 291:F495–F502. https://www.physiology.org/doi/10.1152/ajprenal.00521.2005 10.1152/ajprenal.00521.2005 16609147

[16] Kessel F, Kröger H, Gerlach M, Sradnick J, Gembardt F, Todorov V, Hugo C (2020) A new analysis approach for single nephron GFR in intravital microscopy of mice. F1000Research 9:1372. https://f1000research.com/articles/9-1372/v1 10.12688/f1000research.26888.1 PMC8210690 34290860

[17] Kirkpatrick ND, Chung E, Cook DC, Han X, Gruionu G, Liao S, Munn LL, Padera TP, Fukumura D, Jain RK (2012) Video-rate resonant scanning multiphoton microscopy: an emerging technique for intravital imaging of the tumor microenvironment. Intravital 1:10.4161/intv.21557. http://www.ncbi.nlm.nih.gov/pubmed/24353926 10.4161/intv.21557 PMC3864876 24353926

[18] Leser KH, Osswald H (1985) Maleate induced fall of glomerular filtration rate. Naunyn Schmiedebergs Arch Pharmacol 331:253–259. http://link.springer.com/10.1007/BF00634246 10.1007/BF00634246 4088323

[19] Levine DZ, Iacovitti M, Robertson SJ, Mokhtar GA (2006) Modulation of single-nephron GFR in the db/db mouse model of type 2 diabetes mellitus. Am J Physiol Regul Integr Comp Physiol 290:R975–81. http://www.ncbi.nlm.nih.gov/pubmed/16339386 10.1152/ajpregu.00693.2005 16339386

[20] Lindheimer MD, Lalone RC, Levinsky NG (1967) Evidence that an acute increase in glomerular filtration has little effect on sodium excretion in the dog unless extracellular volume is expanded *. J Clin Invest 46:256–265. http://www.jci.org/articles/view/105528 10.1172/JCI105528 PMC297044 6018763

[21] Lorenz JN (2012). Micropuncture of the kidney: a primer on techniques. Comprehensive physiology.

[22] Luippold G, Mühlbauer B (1998). Dopamine D2 receptors mediate glomerular hyperfiltration due to amino acids. J Pharmacol Exp Ther.

[23] Munger K, Baylis C (1988) Sex differences in renal hemodynamics in rats. Am J Physiol Physiol 254:F223–F231. https://www.physiology.org/doi/10.1152/ajprenal.1988.254.2.F223 10.1152/ajprenal.1988.254.2.F223 3344806

[24] Peterson OW, Gabbai FB, Myers RR, Mizisin AP, Blantz RC (1989) A single nephron model of acute tubular injury: role of tubuloglomerular feedback. Kidney Int 36:1037–44. http://www.ncbi.nlm.nih.gov/pubmed/2601254 10.1038/ki.1989.298 2601254

[25] Peti-Peterdi J, Burford JL, Hackl MJ (2012) The first decade of using multiphoton microscopy for high-power kidney imaging. Am J Physiol Physiol 302:F227–F233. https://www.physiology.org/doi/10.1152/ajprenal.00561.2011 10.1152/ajprenal.00561.2011 PMC3340919 22031850

[26] Petrillo F, Iervolino A, Angrisano T, Jelen S, Costanzo V, D’Acierno M, Cheng L, Wu Q, Guerriero I, Mazzarella MC, De Falco A, D’Angelo F, Ceccarelli M, Caraglia M, Capasso G, Fenton RA, Trepiccione F (2021) Dysregulation of principal cell miRNAs facilitates epigenetic regulation of AQP2 and results in nephrogenic diabetes insipidus. J Am Soc Nephrol ASN.2020010031 10.1681/ASN.2020010031 PMC8259636 33727367

[27] Remuzzi A, Puntorieri S, Battaglia C, Bertani T, Remuzzi G (1990) Angiotensin converting enzyme inhibition ameliorates glomerular filtration of macromolecules and water and lessens glomerular injury in the rat. J Clin Invest 85:541–9. http://www.ncbi.nlm.nih.gov/pubmed/1688888 10.1172/JCI114470 PMC296456 1688888

[28] Remuzzi A, Puntorieri S, Mazzoleni A, Remuzzi G (1988) Sex related differences in glomerular ultrafiltration and proteinuria in Munich-Wistar rats. Kidney Int 34:481–486. https://linkinghub.elsevier.com/retrieve/pii/S0085253815343799 10.1038/ki.1988.206 3199667

[29] Sabbatini M, Esposito C, De Nicola L, Uccello F, Altomonte M, Conte G, Dal Canton A, Andreucci VE (1989) Reversibility of acute cyclosporin nephrotoxicity by dopamine. micropuncture study in the rat. Nephrol Dial Transplant 4:327–333. https://academic.oup.com/ndt/article/1875494/Reversibility 10.1093/oxfordjournals.ndt.a091885 2505181

[30] Sandoval RM, Molitoris BA (2017) Intravital multiphoton microscopy as a tool for studying renal physiology and pathophysiology. Methods 128:20–32. https://linkinghub.elsevier.com/retrieve/pii/S1046202316304121 10.1016/j.ymeth.2017.07.014 PMC5730351 28733090

[31] Schindelin J, Arganda-Carreras I, Frise E, Kaynig V, Longair M, Pietzsch T, Preibisch S, Rueden C, Saalfeld S, Schmid B, Tinevez J-Y, White DJ, Hartenstein V, Eliceiri K, Tomancak P, Cardona A (2012) Fiji: an open-source platform for biological-image analysis. Nat Methods 9:676–682. http://www.nature.com/articles/nmeth.2019 10.1038/nmeth.2019 PMC3855844 22743772

[32] Seri I, Aperia A (1988) Contribution of dopamine 2 receptors to dopamine-induced increase in glomerular filtration rate. Am J Physiol Physiol 254:F196–F201. https://www.physiology.org/doi/10.1152/ajprenal.1988.254.2.F196 10.1152/ajprenal.1988.254.2.F196 2964202

[33] Tonneijck L, Muskiet MHA, Smits MM, van Bommel EJ, Heerspink HJL, van Raalte DH, Joles JA (2017) Glomerular hyperfiltration in diabetes: mechanisms, clinical significance, and treatment. J Am Soc Nephrol 28:1023–1039. https://jasn.asnjournals.org/lookup/doi/10.1681/ASN.2016060666 10.1681/ASN.2016060666 PMC5373460 28143897

[34] Trepiccione F, Soukaseum C, Baudrie V, Kumai Y, Teulon J, Villoutreix B, Cornière N, Wangemann P, Griffith AJ, Byung Choi Y, Hadchouel J, Chambrey R, Eladari D (2017). Acute genetic ablation of pendrin lowers blood pressure in mice. Nephrol Dial Transplant.

[35] Ueda J, Nygren A, Hansell P, Erikson U (1992) Influence of contrast media on single nephron glomerular filtration rate in rat kidney. A comparison between diatrizoate, iohexol, ioxaglate, and iotrolan. Acta Radiol 33:596–9. http://www.ncbi.nlm.nih.gov/pubmed/1449888 1449888

[36] Wright FS, Giebisch G (1972) Glomerular filtration in single nephrons. Kidney Int 1:201–209. https://linkinghub.elsevier.com/retrieve/pii/S0085253815310292 10.1038/ki.1972.30 4671212

[37] Zipfel WR, Williams RM, Webb WW (2003) Nonlinear magic: multiphoton microscopy in the biosciences. Nat Biotechnol 21:1369–77. http://www.ncbi.nlm.nih.gov/pubmed/14595365 10.1038/nbt899 14595365

