# Supplementary figures and images for: Single nephron glomerular filtration rate measured by linescan multiphoton microscopy compared to conventional micropuncture
Source: Pflugers Arch. 2022 Apr 9;474(7):733–41. doi: 10.1007/s00424-022-02686-8 (PMC9192459; doi:10.1007/s00424-022-02686-8)

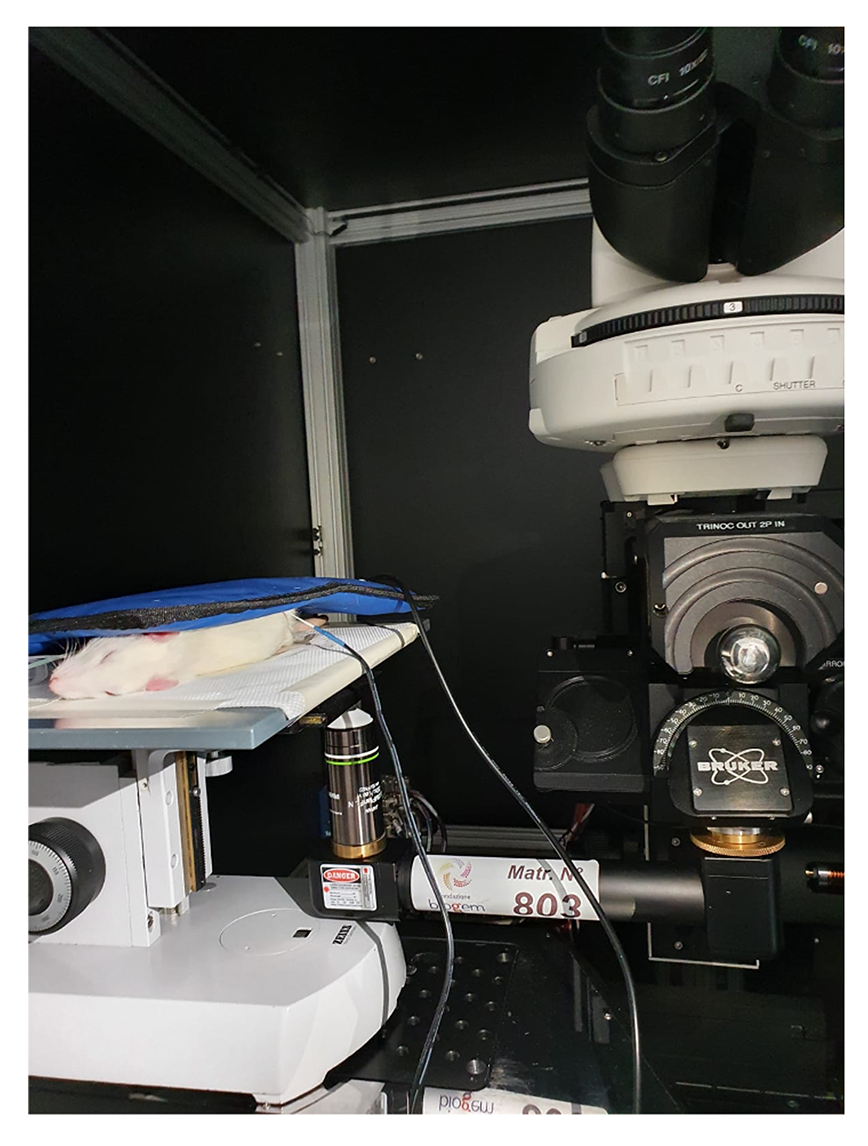

Supplement: Supplementary file 1 — Experimental setup for in vivo MPM imaging of the kidney. Anesthetized animals were surgically prepared for intravital imaging as described in methods. An inverted arm was used to acquire the images in order to minimize the movements due to breathing. A water-immersion 20X NA 1.0 objective was used to image the renal structures. The animal was covered by a warm pad for the entire experimental procedure. (PNG 801 kb) [file 424_2022_2686_Fig4_ESM.png]

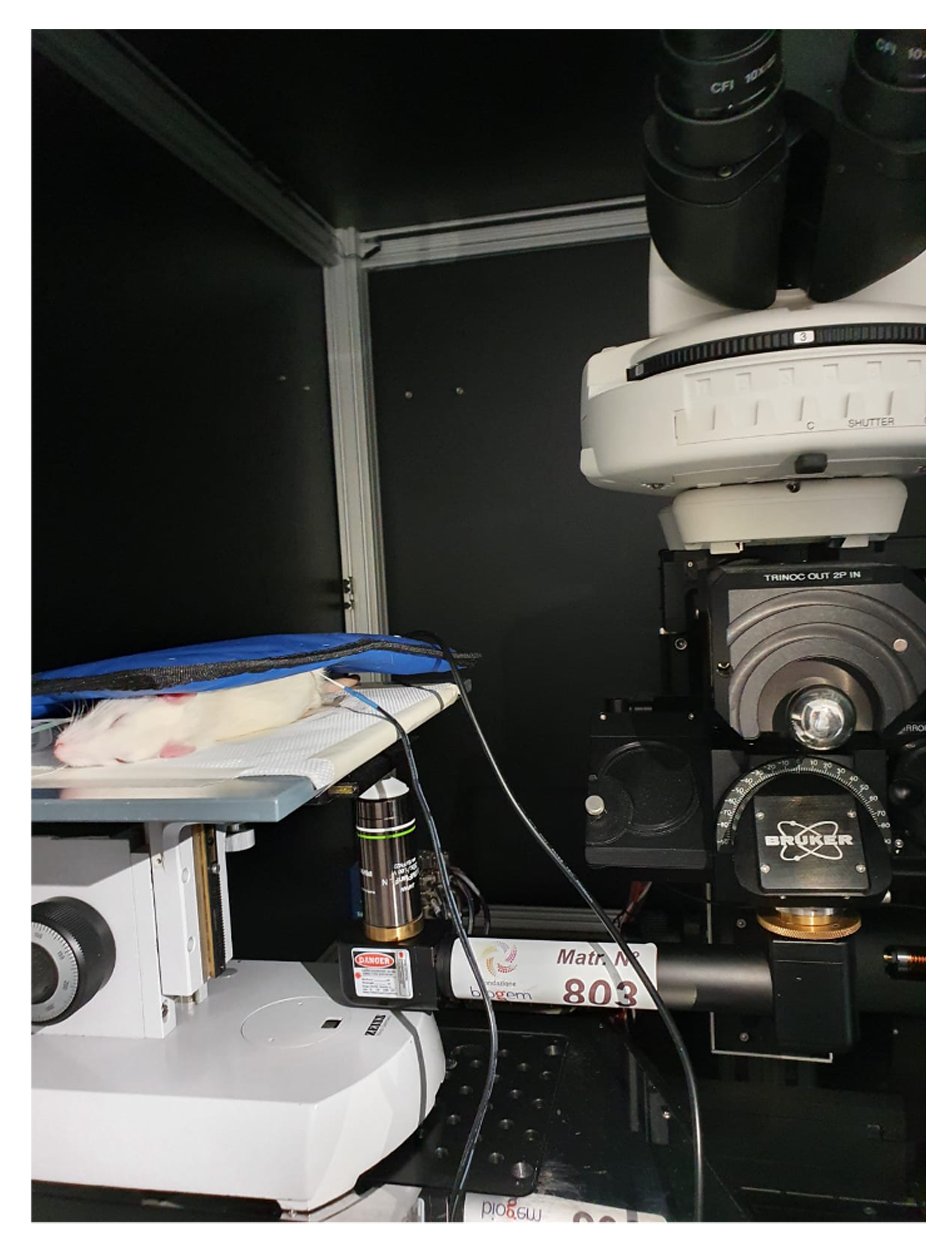

Supplement: Supplementary file 2 — High Resolution Image (TIF 2596 kb) [file 424_2022_2686_MOESM1_ESM.tif]

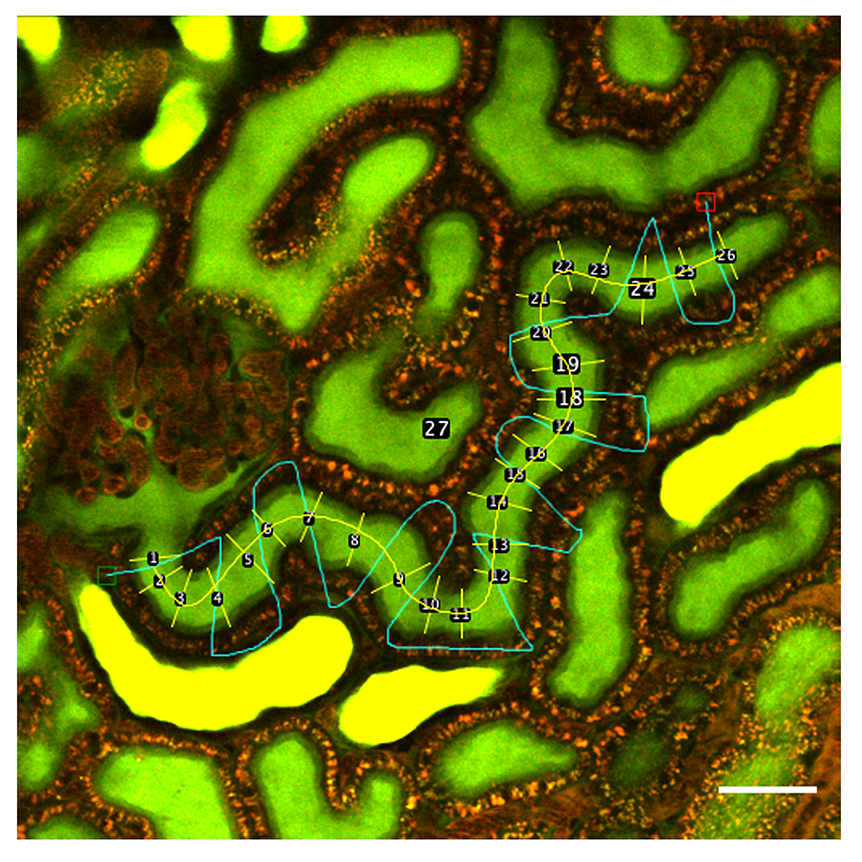

Supplement: Supplementary file 3 — Measurement of tubular length and diameter of S1 proximal tubule. Representative image of the S1 proximal tubule after the linescan acquisition. The tubular length was measured within the two crosses of interest by following the central axis of the lumen. The tubular diameter was measured in several points along the tubule, then all measurements were averaged to measure the SNGFR. Fiji software was used to analyze the images. Scale bar is 50 μm (PNG 991 kb) [file 424_2022_2686_Fig5_ESM.png]

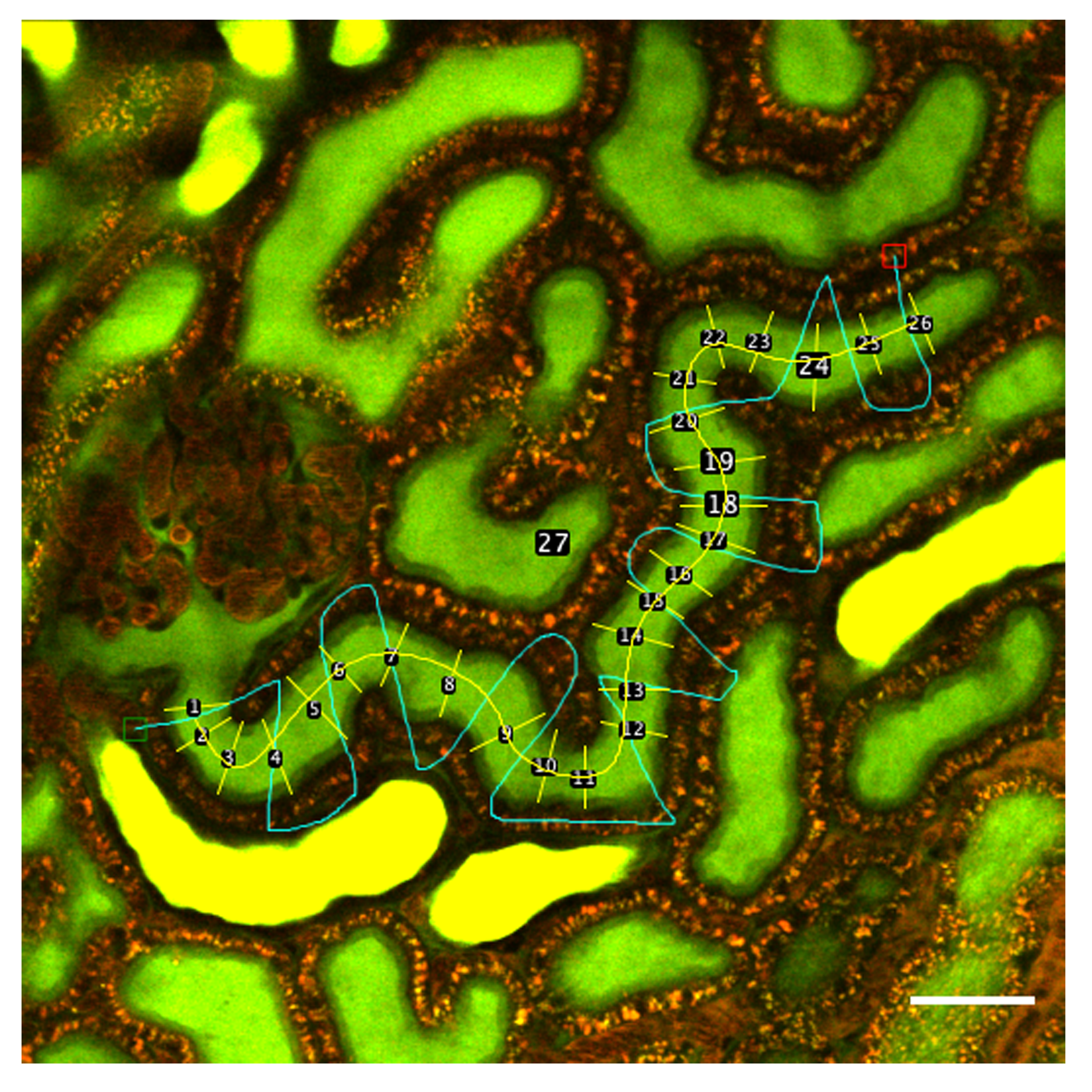

Supplement: Supplementary file 4 — High Resolution Image (TIF 3970 kb) [file 424_2022_2686_MOESM2_ESM.tif]

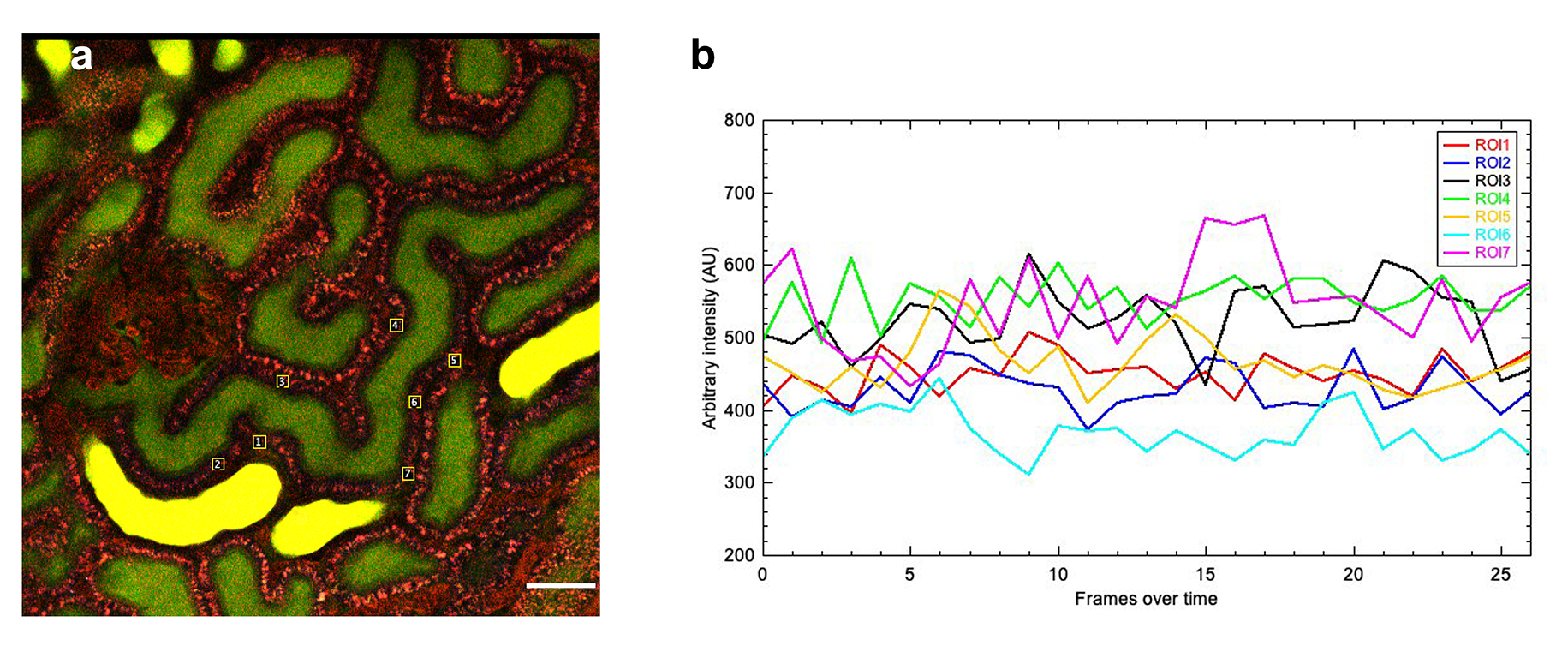

Supplement: Supplementary file 5 — Analysis of intracellular fluorescence during dextran bolus administration. Seven ROIs were selected in the intracellular compartment of S1 proximal tubule (panel a) and the variation of fluorescence intensity expressed as arbitrary unit intensity (AU) was recorded over the time the bolus injection of FITC-dextran 3-5 kDa streamed along the tubule (panel b). Each ROI is represented by a different color. Scale bar is 50 μm. (PNG 1420 kb) [file 424_2022_2686_Fig6_ESM.png]

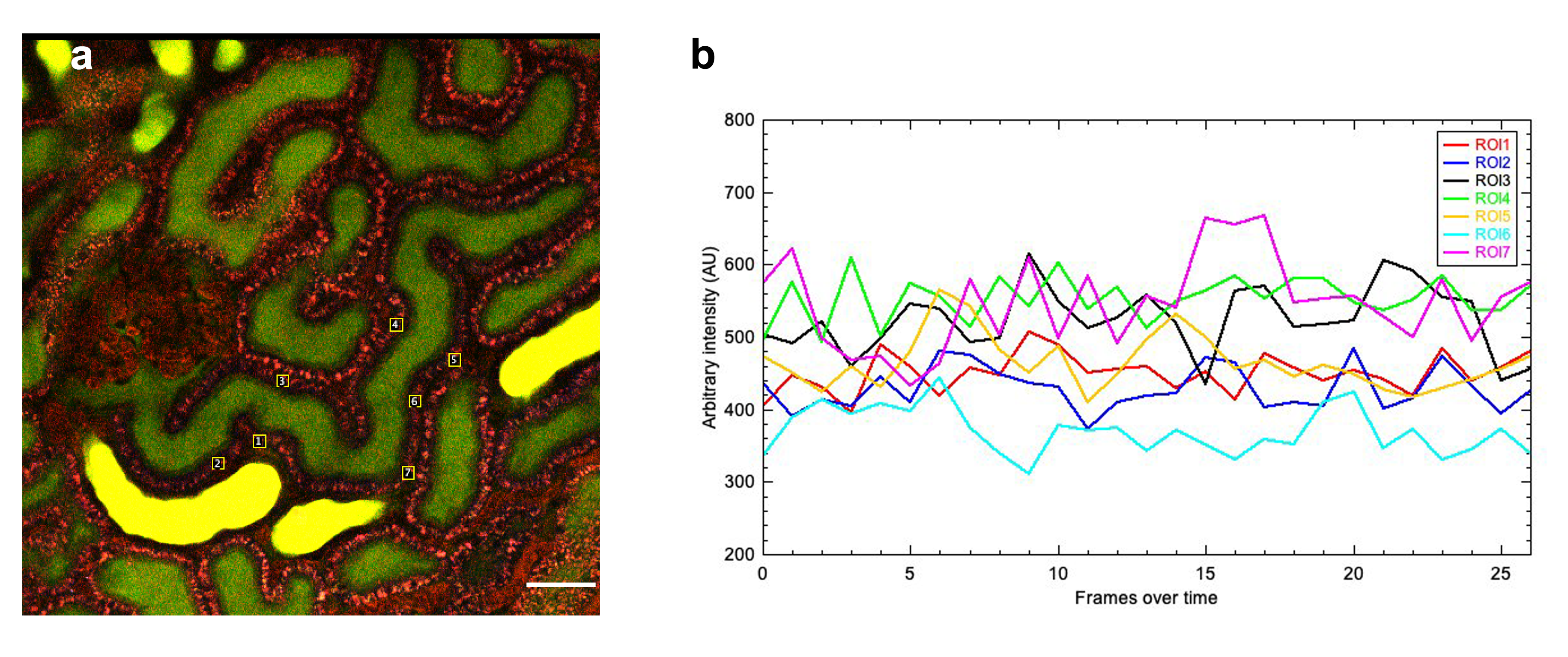

Supplement: Supplementary file 6 — High Resolution Image (TIF 6327 kb) [file 424_2022_2686_MOESM3_ESM.tif]

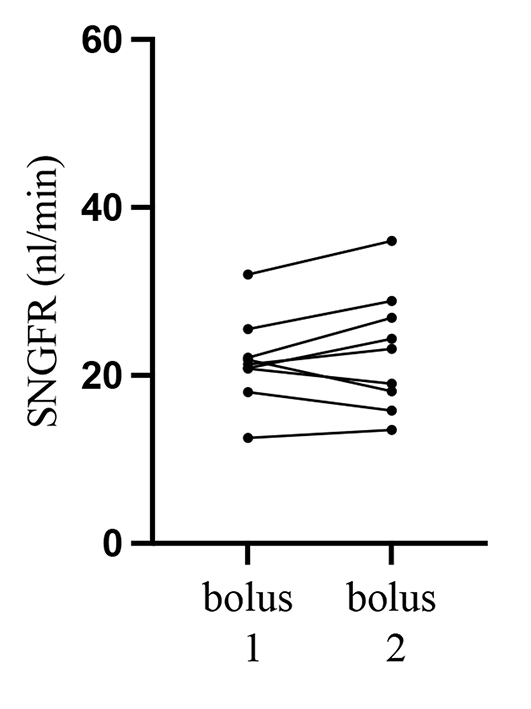

Supplement: Supplementary file 7 — Consistency of the SNGFR over time along the same S1 proximal tubule. SNGFR was measured two consecutive times in nine S1 proximal tubules from different animals. In particular, a second bolus of the fluorescent dye (bolus 2) was performed in the same tubule within 5-10 minutes after the first one was injected (bolus 1). No difference of SNGFR values after bolus 1 and bolus 2 was detected (Paired t test). (PNG 33 kb) [file 424_2022_2686_Fig7_ESM.png]

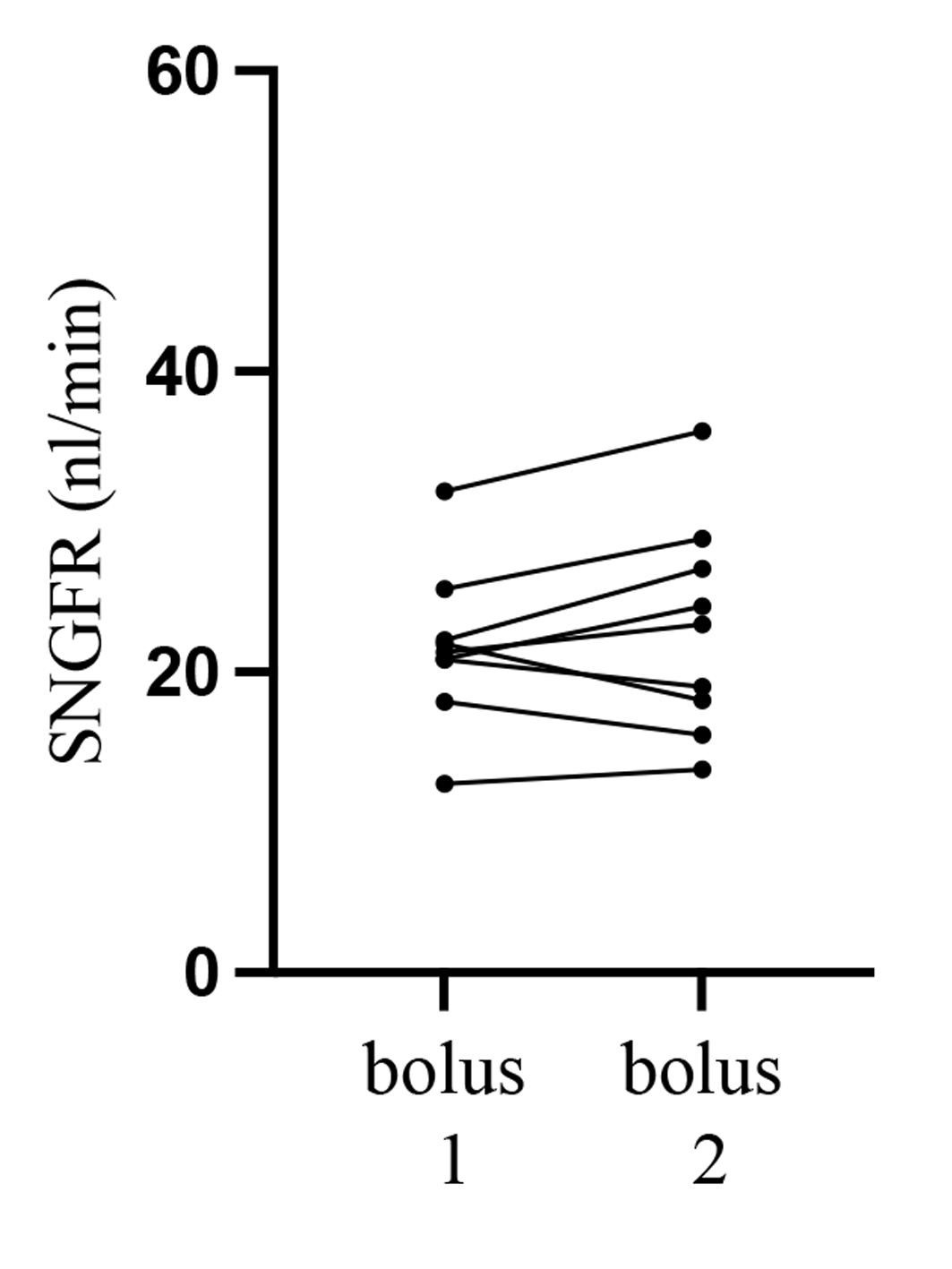

Supplement: Supplementary file 8 — High Resolution Image (TIF 118 kb) [file 424_2022_2686_MOESM4_ESM.tif]

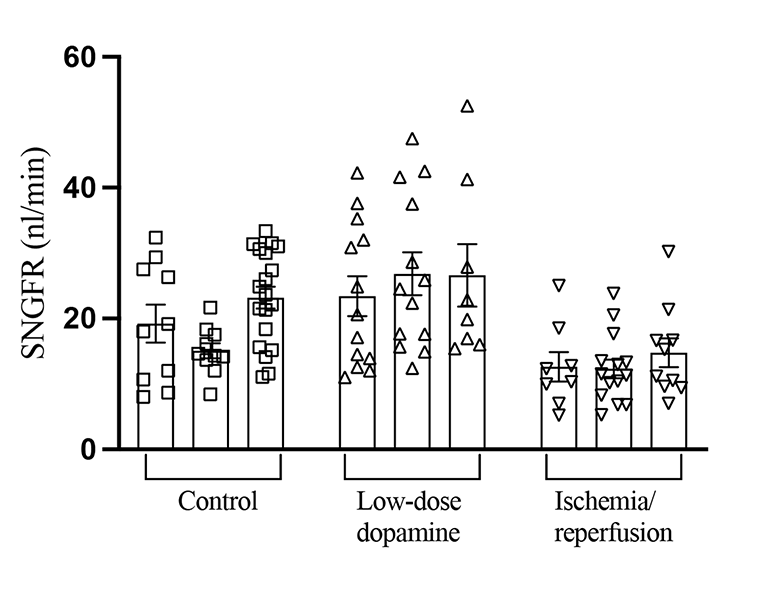

Supplement: Supplementary file 9 — SNGFR per single S1 segment at control, low dose dopamine and IRI. Single data from each S1 segment are plotted. Each bar represents measurements from one experimental rat. Mean values ± standard error are represented. (PNG 53 kb) [file 424_2022_2686_Fig8_ESM.png]

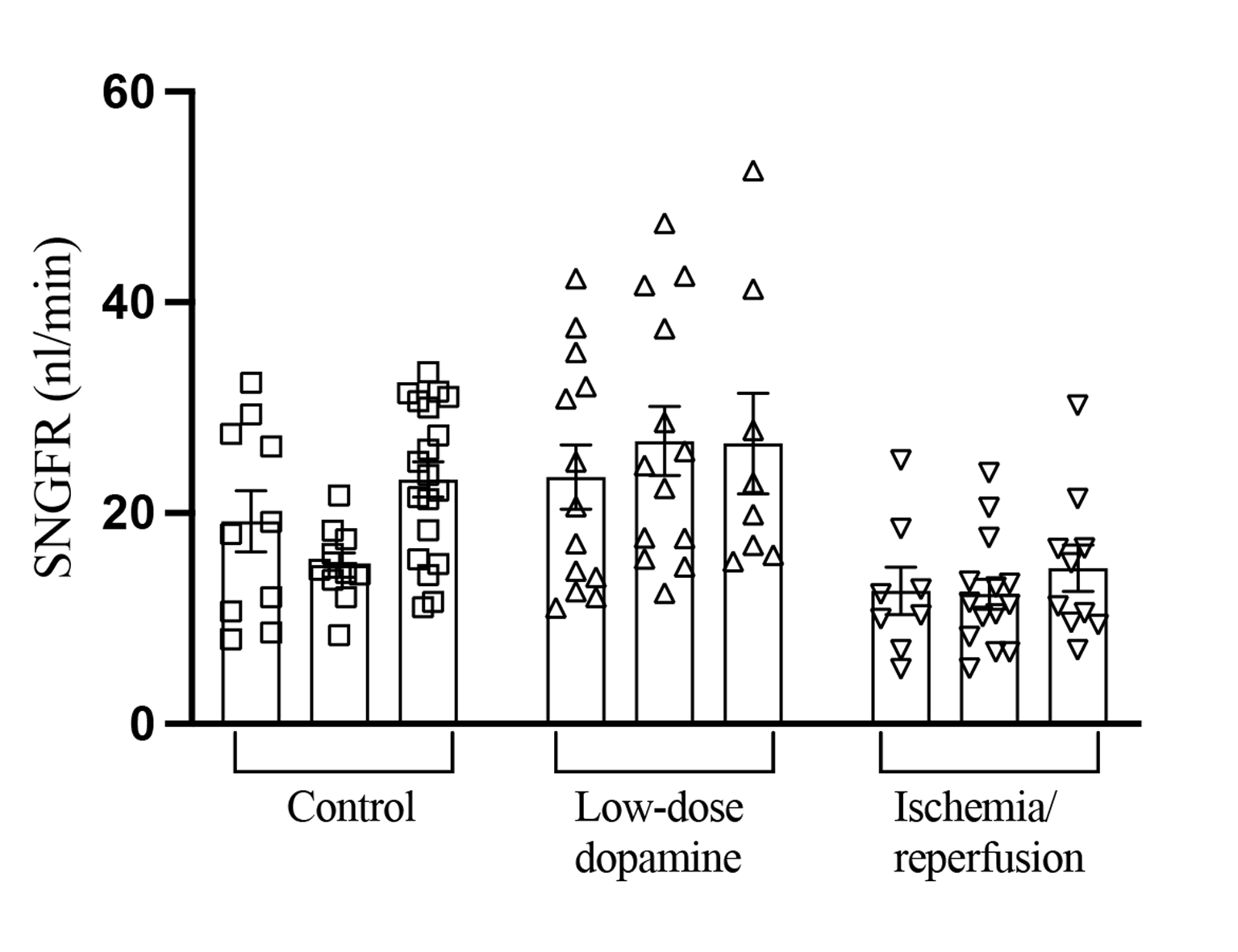

Supplement: Supplementary file 10 — High Resolution Image (TIF 215 kb) [file 424_2022_2686_MOESM5_ESM.tif]
