# Supplementary material for: Single nephron glomerular filtration rate measured by linescan multiphoton microscopy compared to conventional micropuncture
Source: Pflugers Arch. 2022 Apr 9;474(7):733–41. doi: 10.1007/s00424-022-02686-8 (PMC9192459; doi:10.1007/s00424-022-02686-8)
Supplement: Supplementary file 11 — Comparison of the experimental setting used in MPM linescan tool and previous micropuncture studies. The table compares the physiological parameters of rats used for experiments for linescan method and previous micropuncture. In particular, the age (days) and the body weight (g) of the animals and the references are reported. (DOCX 15 kb) [file 424_2022_2686_MOESM6_ESM.docx]

| **Group** | **Method** | **Age (days)** | **Body weight (g)** | **Ref** |
| --- | --- | --- | --- | --- |
| Male control | Micropuncture | ≥60 | 270-345 | [32] |
|  | Linescan | 60-70 | 260-295 |  |
| Female control | Micropuncture | 90-120 | 190-198 | [23] |
|  | Linescan | 70-90 | 170-220 |  |
| Low-dose dopamine | Micropuncture | Not indicated | 170-230 | [29] |
|  | Linescan | 70-80 | 176-197 |  |
| Ischemia/  reperfusion | Micropuncture | Not indicated | 200-260 | [1] |
|  | Linescan | 75-85 | 185-205 |  |
